# Callous-unemotional behaviors and conduct problems in Chinese preschoolers: the moderating roles of surgency and gender

**DOI:** 10.3389/fpsyg.2024.1328345

**Published:** 2024-05-09

**Authors:** Jingjing Zhu, Xin Shu, Zhuyi Li, Yan Li

**Affiliations:** ^1^Shanghai Institute of Early Childhood Education, Shanghai Normal University, Shanghai, China; ^2^Jingan District Anqing Shanghai Kindergarten, Shanghai, China

**Keywords:** callous-unemotional behaviors, conduct problems, surgency, gender, preschool children, China

## Abstract

**Introduction:**

Conduct problems in children are related to callous-unemotional (CU) behaviors. However, results of the relationships between CU behaviors and conduct problems among preschoolers mainly focused on Western countries, no studies have examined whether CU behaviors predict conduct problem in Chinese preschoolers. The primary objective of the current study therefore was to examine the associations between CU behaviors and conduct problems as well as the moderating effects of surgency and child gender in Chinese preschool children.

**Methods:**

The present study randomly selected 2,154 children (1,043 boys, *M*_age_ = 56 months, *SD* = 10.47) from six kindergartens in Shanghai, People’s Republic of China. Mothers rated children’s surgency and teachers reported children’s CU behaviors and conduct problems.

**Results:**

Results demonstrated that CU behaviors were positively associated with conduct problems. Surgency and child gender significantly moderated these associations. Specifically, CU behaviors were positively associated with conduct problems, with a stronger effect found for high levels of surgency. CU behaviors had a positive association with conduct problems, with a stronger effect found for boys.

**Discussion:**

This study indicate that temperament and gender characteristics influence conduct problems in preschoolers who exhibit high levels of CU behaviors. As well, the findings emphasize the significance of considering the meaning and implication of CU behaviors in Chinese culture.

## Introduction

Children who demonstrate high levels of callous-unemotional (CU) behaviors have lower levels of remorse and empathy, a deficit in emotional expression, and a low concern for their own performance (Frick and White, 2008; Frick et al., 2014; Waller et al., 2020). CU behaviors were linked to deficiency in recognizing negative emotions of others (Rehder et al., 2017), fearlessness to threat and low affiliation to others (Waller and Wagner, 2019; Domínguez-Álvarez et al., 2021). In recent years, there has been a gradual increase in studies exploring CU behaviors among preschool children (Waller et al., 2012; Waller et al., 2015, Song et al., 2016, Kimonis et al., 2019, Waller and Wagner, 2019, Domínguez-Álvarez et al., 2021). These studies have shown that CU behaviors are associated with children’s negative developmental outcomes (Song et al., 2016; Waller et al., 2017). For example, CU behaviors were not only positively correlated with children’s poor peer relationships (Waller et al., 2017), but also with aggressive behaviors in middle to late childhood (Waller et al., 2017; Obando et al., 2022). CU behaviors as a risk factor help to identify children who exhibit persistent and severe conduct problems (Longman et al., 2016; Song et al., 2016; Waller et al., 2016, 2017). However, these studies have been limited largely to Western countries (Frick et al., 2014). In China, research on CU behaviors is in its nascent stages, with samples predominantly concentrated on adolescents and college students (Fang et al., 2020; Zhang et al., 2023). Only a few studies on CU behaviors have been conducted with Chinese preschool children (Deng et al., 2016; Zhang et al., 2021; Cao et al., 2023; Zhu et al., 2023a,b). Different from the individualism in Western cultures, Chinese is the collectivism cultures whose communication style between persons tends to be indirect and non-expressive rather than direct and expressive (Wang and Liu, 2010). There, it may lead to higher scores on the “Unemotional” scale of Inventory of Callous-Unemotional Traits among Chinese children (ICU; Frick et al., 2003; Fung et al., 2009; Sng et al., 2020). Considering the negative influence of CU behaviors on preschool children’s development outcomes and culture differences, it was necessary to explore CU behaviors among Chinese preschool children. The first purpose of current study was to investigate the relationships between CU behaviors and conduct problems in Chinese preschoolers. Besides, the developmental process about how CU behaviors lead to conduct problems, and how other child-level factors influence these processes are unclear. Thus, the second goal of current study was to examine the potential moderating role surgency and child gender in the associations between CU behaviors and conduct problems.

### CU behaviors and conduct problems

Researchers have found children with high levels of CU behaviors tend to prefer dangerous and novel activities (Frick et al., 2003), exhibit deficiency in recognizing negative emotions of others (Rehder et al., 2017), and low levels of empathy and guilt (Frick and White, 2008). These characteristics seem to increase the risk of children developing severe and chronic conduct problems (Frick et al., 2014). Numerous studies indicate that CU behaviors were risk factors that influence children to exhibit severe and stable conduct problems throughout middle and late childhood (Frick et al., 2014; Waller et al., 2016, 2017). For example, CU behaviors rated by mothers at age 3 independently predicted persistently high levels of aggression rated by teachers from ages 6 to 12 (Willoughby et al., 2014). Waller and colleagues discovered in several longitudinal studies of preschool children that CU behaviors in early childhood significantly predicted their conduct problems in school age after controlling for externalizing problems in the preschool years (Waller et al., 2015, 2016, 2017). Furthermore, a meta-analysis of a sample of 24–80 months preschool children found a significant positive association between CU behaviors and conduct problems with a large effect size ( *r* = 0.39, *p* < 0.001) (Longman et al., 2016). Taken together, it was evident that early CU behaviors was a significant predictor for identifying children who were likely to endure severe and persisting conduct problems throughout childhood. However, most studies on CU behaviors and conduct problems in preschool children have been conducted with samples at risk for conduct problems (Waller et al., 2015, 2016, 2017; Song et al., 2016), and it needs to be verified whether the findings can be extrapolated to typically developing children. Some studies in China have examined the relationships between CU behaviors and rule-breaking behaviors in school-aged children (Ma et al., 2022) and the relationships between CU behaviors and school bullying in middle school students (Zhang et al., 2023), no studies have examined how CU behaviors affect conduct problems in Chinese preschoolers. In order to further illuminate early risk factors for conduct problems and potential targets for minimizing the likelihood of children developing more severe types of conduct problems later (Kimonis et al., 2019), present study examined the relationships between CU behaviors and conduct problems among typically developing children aged 3–6 years in China. Considering parents may have more difficulty objectively evaluating children’s negative behaviors due to subjective emotional factors, we used teacher reports to assess children’s CU behaviors and conduct problem in current study, consistent with previous research (Wagner et al., 2020). Based on the previously described findings, we hypothesized that CU behaviors would be positively associated with conduct problems in Chinese preschool children.

Although the extant studies suggest that CU behaviors may serve as a risk factor for conduct problems, it remains unknown how conduct problems are influenced by CU behaviors and how child-level factors moderate those processes. Some studies have found that child-level features, such as a fearful/inhibited temperament, theory-of-mind, autonomic functioning and executive function, moderated the relationships between CU behaviors and conduct problems in preschool children (Song et al., 2016; Waller et al., 2017; Wagner et al., 2018). These findings indicate that it is important to examine co-occurring child features when trying to understand how the developmental processes by which CU behaviors affect conduct problems. Surgency, a temperamental feature, and gender are important individual features of child. Present study aims to explore the moderating role of surgency and gender between CU behavior and conduct problems in young children. According to the multi-deterministic perspectives on psychopathology (Cicchetti, 2014) and biopsychosocial framework (Calkins et al., 2013), the associations between CU behaviors and conduct problems may be exacerbated or buffered by child biological characteristics, such as the gender and surgency.

### The moderating role of surgency

Surgency is an important component of temperament and it reflects children’s levels of sociality, impulsivity, activity, and positive emotions in response to challenging situations (Rothbart, 2012; Garon-Carrier et al., 2022). Children with high levels of surgency have high social initiative and low shyness, generally disregard social rules and boundaries (Garon-Carrier et al., 2022), and exhibit impulsive, risk-taking, and sensation-seeking behaviors (Rothbart, 2012). Research has revealed that high levels of surgency were associated with more conduct problems in children (Rothbart et al., 2001; Berdan et al., 2008; Scheper et al., 2017; Behrendt et al., 2020; del Puerto-Golzarri et al., 2022). For example, surgency assessed at age 4.5 was positively associated with externalizing behaviors 1 year later (Berdan et al., 2008). Higher levels of surgency were linked to more externalizing problem behaviors in clinically referred children (Scheper et al., 2017). In a sample of 3–6 years old Chinese preschool children, surgency was positively associated with aggressive behaviors (Liu et al., 2020). Thus, surgency may be a risk factor for children with conduct problems.

According to the Risk-Enhancing Model (REM), when one risk factor and another risk factor are cumulative, the adverse effects on individuals will exceed the simple sum of the two risk factors (Wang et al., 2012). Based on this model, surgency (a risk factor) may exacerbate the effect of CU behaviors (a risk factor) on young children’s conduct problems. Children with high CU behavior were at risk for conduct problems due to their lack of empathy (Waller et al., 2015), which made it difficult for them to relate their adverse acts toward others with the hurtful emotions experienced by others (Blair, 1995). Due to their high levels of impulsivity and social initiative, which could lead to more conflicts in interpersonal relationships, it was believed that children with high levels of surgency were more likely to have behavioral issues (Behrendt et al., 2020; Liu et al., 2020). Together, children who have dual risk—high levels of surgency and high levels of CU behaviors—may show more conduct problems compared to those who either exhibit high levels of surgency or high levels of CU behaviors. Previous research has confirmed the interaction between CU behaviors and temperament components. For example, high levels of fearlessness, a component of temperament, could exacerbate the relationship between children’s CU behaviors and conduct problems (Song et al., 2016). However, research has yet to be conducted to determine whether the interplay of CU behaviors and surgency predicts a higher likelihood of conduct problems. In the current study, we hypothesized that the relationships between CU behaviors and conduct problems would be significant in both children with high levels of surgency and low levels of surgency, but with a stronger effect found for high levels of surgency than low levels of surgency.

### The moderating role of gender

Gender plays an important role in children’s conduct problems. Many longitudinal studies have found significantly higher levels of conduct problems in boys compared to girls (Berkout et al., 2011; Paz et al., 2021). In a sample of Chinese preschoolers, boys had higher levels of conduct problems than girls (Bai et al., 2022).

According to Relational Theory, females are more eager than males to develop and maintain positive relationships with others (Miller, 1976; Miller and Stiver, 1997). Girls with high levels of CU behaviors may be more inclined than boys to suppress conduct problems, such as aggression so that these behaviors do not damage their relationships with others. In contrast, for boys, their relatively low desire to establish relationships (Miller, 1976; Miller and Stiver, 1997) prompts them to engage in more conduct problems driven by CU behaviors due to their unconcern about whether these behaviors problems would damage their relationships with others. Moreover, traditional gender role expectations require females to be demure and ladylike, while males to be brave (Eagly, 1987). Influenced by social expectations, girls are more likely to engage in social aggression, rather than overt forms of aggression (Davies, 2014; Leff et al., 2024). Thus, girls with high levels of CU behaviors may show less conduct problems. Besides, compared to girls, society is more tolerant to boy’s aggressive and rule-breaking behaviors, which are seen as markers of masculinity (Wang et al., 2020). Thus, boys with high levels of CU behaviors may not be to restrain themselves from showing such behaviors under the influence of gender stereotypes and social expectations. Gender may act as a moderator in the relationships between CU behaviors and conduct problems.

However, few studies have explored gender differences in the relationships between CU behaviors and conduct problems in young children (Waller et al., 2016, 2017). In a sample of American college students, it was found that higher levels of CU behaviors were associated with more physical aggression in both boys and girls, with a notably stronger predictive effect observed in boys (Caiozzo et al., 2016). It is necessary to know whether there are similar results in a sample of preschoolers in China. We hypothesized that CU behaviors were positively related with conduct problems with a strong effect for boys than girls.

### The current study

**Figure 1:**
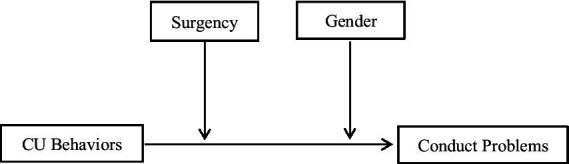
Relationship path map of CU behaviors, surgency, gender, and conduct problems.

The first goal of this study was to examine the association between CU behaviors and conduct problems. We hypothesized that CU behaviors were positively related to preschool children’s conduct problems. The second purpose was to examine the role of surgency in moderating the links between CU behaviors and conduct problems. We hypothesized that CU behaviors would be positively associated with conduct problem with a stronger effect found for high levels of surgency than low levels of surgency. Finally, we also aimed to examine the gender difference of the association between CU behaviors and conduct problems. We hypothesized that the relationship between CU behaviors and conduct problems was stronger in boys than in girls. The research hypotheses were shown in Figure 1.

## Method

### Participants

In this study, the participants consisted of 2,154 preschoolers (1,043 boys, 1,111 girls; *M*_age_ = 56 months, *SD* = 10.47) in 93 classes of 6 kindergartens in Shanghai, People’s Republic of China. We randomly selected 6 kindergartens in Shanghai. Children with serious health problems, mental retardation, and pervasive developmental disorders were excluded in current study. We also excluded from our study children whose mothers suffered from physical or mental ailments or reading disorders. In each kindergarten, all children who met our inclusion criteria were invited to participate in the study, and their participation was voluntary. In China, kindergarten includes three levels: junior class (3–4 years-old), middle class (4–5 years-old), and senior class (5–6 years-old). Among the participants, 738 children are from junior class (age range = 3.00–4.83 years, M_age_ = 3.69, SD = 0.29), 688 children are from middle class (age range = 3.50–5.92 years, M_age_ = 4.58, SD = 0.30) and 728 children are from senior class (age range = 4.33–6.50 years, M_age_ = 5.67, SD = 0.31). China is a multi-ethnic country, with Han ethnicity as the main group, accounting for 92%. In this study, the ethnicity of all children was Han ethnicity. As for caregivers’ educational levels, about 21.5% of mothers and 24.1% of fathers were at high school level or below, about 58.4% of mothers and 54.2% of fathers had a bachelor’s degree, 16.5% of mothers and 21.6% of fathers had a post-graduate degree or above. The mean of mother’s and father’s education levels were taken as an indicator of parental education level (Hill et al., 2004).

### Measures

#### Callous-unemotional behavior

The Inventory of Callous-Unemotional Traits (ICU; Frick et al., 2003) was used to assess teacher-reported CU behavior. We use translation and back-translation method to develop the Chinese version of the ICU (Zhu et al., 2023a). It consists of 21 out of the original 24 items and taps into three factors: callousness (9 items, e.g., “feels bad or guilty when he/she has done something wrong”), uncaring (9 items, e.g., “does not care about doing things well”) and unemotional (3 items, e.g., “does not show emotions”). The three factors model has a good fit indices, *χ^2^* = 801.97, *df* = 166, *χ^2^/df* = 4.83, *p* < 0.001, CFI = 0.95, TLI = 0.93, RMSEA = 0.05. Items for each scale are rated on a 4-point Likert scale from 0 (not true at all) to 3 (definitely true). ICU total scores are calculated by averaging all subscale items. Higher scores are indicative of an increased presence of CU behavior. In the present study, the internal consistencies of total ICU general factor, callous factor, uncaring factor and unemotional factor is.92, 0.89, 0.91 and.65, respectively, which are considered an acceptable.

#### Conduct problem

Teachers also completed the Chinese version of the Strengths and Difficulties Questionnaire (SDQ; Goodman, 1997; Du et al., 2008). The 25-item scale consists of five subscales: Prosocial behavior (5 items), Emotional symptoms (5 items), Conduct problems (5 items), Hyperactivity/Inattention (5 items), and Peer problems (5 items). Of particular interest was the conduct problem scale (Cronbach’s α = 0.72, e.g., “Often fight with other children or bully them”). All items were scored 0 for “not true,” 1 for “somewhat true,” and 2 for “certainly true.” A higher score indicates a higher level of conduct problems. The Chinese version of the SDQ we used has been reported to be reliable and effective (Du et al., 2008).

#### Surgency

Mothers completed the Child Behavior Questionnaire-Short Form (CBQ-SF, Putnam and Rothbart, 2006). The CBQ-SF includes 36 items and consists of effortful control, negative affect, and surgency subscales. Of particular interest for the present study was surgency subscales (12 items, Cronbach’s α = 0.64, e.g., “Likes going down high slides or other adventurous activities”). The CBQ-SF were rated on a seven-point Likert scale, ranging from 1 = extremely untrue of your child to 7 = extremely true of your child, with higher scores reflecting a higher level of surgency. The questionnaire has been used on Chinese children and has good reliability and validity (Zhou et al., 2010).

#### Procedures

The Research Ethics Committee of the host institution approved the current study. We acquired written consent from parents and teachers as well as verbal assent from preschoolers. Nearly at the same time, teachers completed questionnaires to assess children’s CU behaviors and conduct problems and children’s mothers completed the Child Behavior Questionnaire-Short Form. To ensure teacher’s familiarity with the children, variables of CU behaviors and conduct problems were assessed during the spring semester. Teachers and mothers were not given any rewards for participating.

### Statistical analysis

For data analysis, SPSS 26.0 and the PROCESS macro for SPSS were utilized (Hayes, 2018). Preliminary analyses include descriptive statistics and Pearson correlations among all major study variables. Next, we used a series of hierarchical regression analyses to examine whether surgency and gender moderated the effect of CU behaviors on conduct problems (two-way interaction) after centering all continuous predictor variables (Aiken and West, 1991). Specifically, we tested two-way interaction with child surgency and child gender interaction term in two separate regression model. For the model involving child surgency, the covariates child age and parental education level were input in step 1. Step 2 involved entering CU behaviors and surgency as main effects. To predict conduct problems, CU behaviors × Surgency were added in Step 3. For the model involving child gender, the covariates child age, gender and parental education level were input in step 1. Step 2 involved entering CU behaviors and gender as main effects. To predict conduct problems, CU behaviors ×Gender were added in Step 3.

When results revealed a significant interaction, a simple slope analysis was conducted. We probed the significant interactions between CU behaviors and conduct problems at high (+1 SD and + 2 SD above the mean) and low (−1 SD and − 2 SD below the mean) surgency levels. We plotted the significant interactions between CU behaviors and conduct problems for boys and girls (Aiken and West, 1991). The simple slopes for the 2-way interactions were both tested and plotted in the context of the full sample.

## Results

### Preliminary analyses

Table 1 displayed descriptive statistics and correlations of all study variables. Child age was positively associated with surgency (*r* = 0.05, *p* < 0.05), and negatively associated with CU behaviors (*r* = −0.27, *p* < 0.001), and conduct problems (*r* = −0.12, *p* < 0.001). Parental education was positively associated with conduct problems (*r* = 0.05, *p* < 0.05), and negatively associated with surgency (*r* = −0.06, *p* < 0.05). As expected, higher CU behaviors were significantly associated with higher levels of conduct problems (*r* = 0.57, *p* < 0.001). Surgency was significantly positively associated with conduct problems (*r* = 0.11, *p* < 0.001). As such, both child age and parental education were controlled as control variables in the main analyses. The skewness and kurtosis of CU behaviors, conduct problem, and surgency fell within the acceptable range (i.e., skewness < |2.0| and kurtosis < |7.0|; Hancock and Mueller, 2010).

**Table 1:** Descriptive statistics and inter-correlations among main study variables (*N* = 2,154).

	1	2	3	4	5	6
1.Gender	1					
2.Child age	−0.01	1				
3.Parental education	−0.03	0.02	1			
4.CU behaviors	0.21^***^	−0.27^***^	0.03	1		
5.Conduct problems	0.22^***^	−0.12^***^	0.05^*^	0.57^***^	1	
6.Surgency	0.08^***^	0.05^**^	−0.06^*^	0.01	0.11^***^	1
*M*	-	55.75	1.95	0.77	0.26	4.23
*SD*	-	10.47	0.58	0.54	0.35	0.73
Skewness	-	0.12	0.06	0.61	1.76	0.02
Kurtosis	-	−1.22	−0.58	−0.11	2.95	0.36

CU, callous-unemotional; Gender: male = 1, female = 0.***p < 0.001, **p < 0.01, ^*^p < 0.05.

### Moderating effect analysis

Tables 2, 3 illustrated the results of the hierarchical regression models. The next analyze was to explore the potential moderating effect of surgency and gender on the links between CU behaviors and conduct problems.

**Table 2:** Moderating effects of CU behaviors and surgency on conduct problems.

		*β*	SE	*t* value	95% CI	*R*^2^	ΔR^2^	ΔF
Step 1	Child age	−0.12^***^	0.00	−5.52	[−0.01, −0.00]			
	Parental education	0.05^*^	0.01	2.53	[0.01, 0.06]			
Step 2	CU behaviors	0.56^***^	0.01	29.85	[0.34, 0.38]			
Step 3	Surgency	0.09^***^	0.01	5.02	[0.03, 0.06]			
	CU behaviors × Surgency	0.05^**^	0.02	2.95	[0.02, 0.07]	0.347	0.003	8.691^**^

CU, callous-unemotional.***p < 0.001, ^*^p < 0.05.

**Table 3:** Moderating effects of CU behaviors and gender on conduct problems.

		*β*	SE	*t* value	95% CI	*R*^2^	ΔR^2^	ΔF
Step 1	Child age	−0.11^***^	0.00	−5.40	[−0.01, −0.00]			
	Parental education	0.05^*^	0.03	2.18	[0.00, 0.05]			
Step 2	CU behaviors	0.56^***^	0.01	29.72	[0.34, 0.38]			
Step 3	Gender	0.10^***^	0.02	5.65	[0.05, 0.10]			
	CU behaviors × Gender	0.15^***^	0.02	5.87	[0.09, 0.18]	0.347	0.010	34.485^***^

CU, callous-unemotional.^***^p < 0.001, ^**^p < 0.01, ^*^p < 0.05.

### Interactions between CU behaviors and surgency

**Figure 2:**
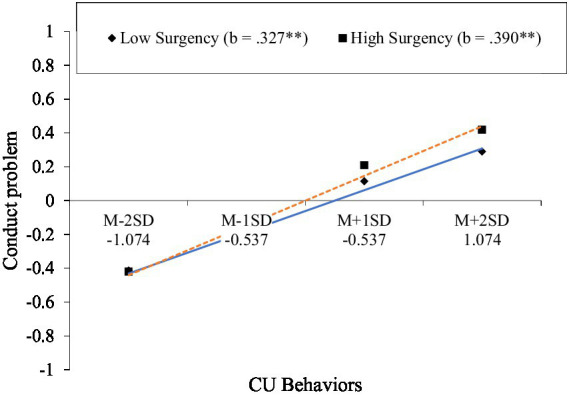
Surgency moderates the relationship between CU behaviors and conduct problems. ^***^p < 0.001; ^**^p < 0.01; ^*^p < 0.05.

As shown in Table 1, controlling for child age and parental education, there was a significant interaction between CU behaviors and surgency predicting conduct problems (*β* = 0.05, SE = 0.02, *t* = 2.95, *p* < 0.01). Following the recommendations of Aiken and West (1991), we tested and plotted simple slopes at 1 SD below (low) and 1 SD above (high) the mean of surgency in order to unravel the interaction effect. Results are illustrated in Figure 1. The findings revealed that CU behaviors had a positive association with conduct problems for children, with a stronger effect found for those with high levels of surgency (*b* = 0.39, SE = 0.02, *t* = 24.48, *p* < 0.01) compared to those with low levels of surgency (*b* = 0.33, SE = 0.02, *t* = 20.13, *p* < 0.01) (see Figure 2).

### Interactions between CU behaviors and gender

**Figure 3:**
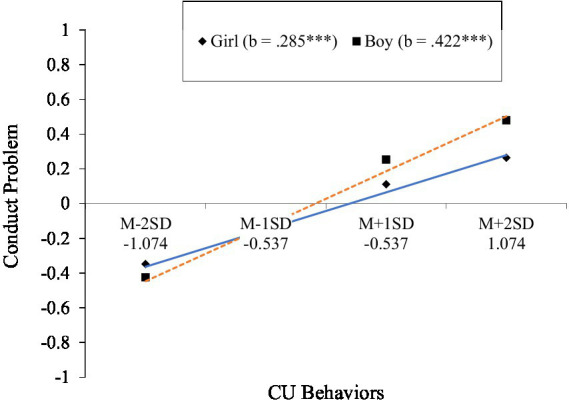
Gender moderates the relationship between CU behaviors and conduct problems.

As shown in Table 1, controlling for child age and parental education, CU behaviors significantly interacted with gender in predicting conduct problems (*β* = 0.15, SE = 0.02, *t* = 5.87, *p* < 0.001). Simple slope analyses revealed that, a significant CU behaviors × gender interaction effects were also found for the prediction of conduct problems. The results revealed that CU behaviors had a positive association with conduct problems, with a stronger effect found for boys (*b* = 0.42, SE = 0.02, *t* = 26.42, *p* < 0.001) than girls (*b* = 0.29, SE = 0.02, *t* = 16.30, *p* < 0.001) (see Figure 3).

## Discussion

The present study firstly examined the relationship between CU behaviors and conduct problems and the moderating role of both surgency and child gender in a sample of Chinese preschoolers. The results showed that CU behaviors is a risk factor for conduct problems, and surgency moderates the link between CU behaviors and conduct problems. Moreover, the findings showed that the link between CU behaviors and conduct problems was moderated by gender, with a stronger risk effect found for boys than for girls. The results and the corresponding implications for future research are discussed as follows.

First, we found that CU behaviors were positively associated with conduct problems in typically developing preschool children despite controlling for child age and parental education. It is in line with our hypothesis and consistent with Western samples (Longman et al., 2016; Waller et al., 2016). These findings suggest that measuring children’s CU behavior contributes to identify children with conduct problems and most in need of intervention.

Second, it is first to consider the role of surgency in the relationship between CU behaviors and conduct problems in preschoolers in China. The results showed that surgency moderated the association between CU behaviors and conduct problems in Chinese preschool children after controlling for child age and parental education. Specifically, CU behaviors had a positive association with conduct problems in children, with a stronger effect found for high levels of surgency than low levels of surgency. This result supported our hypothesis and validated the Risk-Enhancing Model (Wang et al., 2012).

Children with high levels of CU behaviors lacked the ability to recognize and respond to negative emotional cues from others in interpersonal interactions (Frick et al., 2003; Sharp et al., 2006; Díaz-Vázquez et al., 2024), which may result in difficulty understanding others’ sadness or fear and continuously exhibited aggressive behaviors toward others. Children with high levels of surgency may develop more aggression, rule-breaking, and other externalizing problems due to impulsivity, sensation seeking for adventure and exciting situations in more interpersonal interactions. The combination of higher levels of CU behaviors and higher levels of surgency could predict more externalizing behavior problems. Young children with high levels of CU behaviors, high social initiative and impulsive temperament are at higher risk for conduct problems. These findings suggest parents and educators need to be particularly concerned about young children with high levels of CU behavior who are outgoing, impulsive, and risk-seeking and guide young children to follow social behavior norms.

Third, we found that gender played a moderating role in the association between CU behaviors and conduct problems in preschool children, with boy’s CU behaviors being stronger predictors of conduct problems compared to girls. The results are consistent with the findings of Caiozzo et al. (2016) and enrich the results at the preschool stage. For boys, conduct problems including aggression and rule-breaking, are markers of masculinity under the influence of gender stereotypes (Wang et al., 2020), while girls show considerable restraint in externalizing problem behavior in order to conform to sociocultural stereotypes and moral evaluations. Therefore, boys with high levels of CU behaviors would exhibit more conduct problems than girls. Furthermore, the results support the relational theory (Miller, 1976; Miller and Stiver, 1997), suggesting that girls may tend to suppress their undesirable behaviors due to the need to maintain interpersonal relationships. In contrast, for boys, they may tend to show more conduct problems driven by CU behaviors because of their relatively low desire to establish relationships (Miller, 1976; Miller and Stiver, 1997). At the same time, young children with high levels of CU behaviors perceived and responded less to negative emotional cues from others, and empirical research suggests that girls are more attentive to the emotions of others compared to boys (Simon and Nader-Grosbois, 2021). Thus, girls may perceive negative emotional cues from others to a greater degree than boys leading to the weakening effect of girl’s CU behaviors on conduct problems. Notably, teachers may be implicitly influenced by child gender when evaluating CU behaviors and conduct problems (Kim and Chang, 2019). Specifically, teachers may be inclined to give higher scores to girls even though boys and girls may exhibit equal levels of CU behavior and conduct problems. In conclusion, there is a need to replicate the study in different samples in the future and to use multiple methods to examine gender differences in the relationship between CU behaviors and conduct problems in preschool children. The results of this study enlighten parents and educators to focus on conduct problems in boys with high levels of CU behaviors, change gender stereotypes of boys during education and parenting, and guide them to understand the feelings of others and develop pro-social behaviors.

There remained the following research limitations need to be considered. First, this is a cross-sectional study. Thus, the data might not imply any causal relationships or bidirectional relationships. That is, it is not clear whether CU behaviors cause more conduct problems, or if conduct problems lead to increased CU behaviors, or if reciprocal effects are present. Besides, while this study provides evidence for associations between CU behaviors, surgency, gender and high levels of conduct problems, it does not shed light on the mechanisms behind these relationships. A multi-informant, multi-method, longitudinal approach is needed to determine the nature and direction of these relationships and to identify mechanisms explaining the link between CU behaviors, surgency, gender and conduct problems. Second, the sample of current study was recruited from the Shanghai region of China, and it needs to be verified whether the findings can be generalized to preschoolers in other regions of China, especially remote rural areas. Furthermore, CU behaviors and conduct problems of preschoolers were solely relied on teacher reports, which could inflate their associations due to common method variance. Therefore, to reduce the problem of common variance and provide a more comprehensive picture, future studies should consider measuring these variables using multi-informant methods to collect information from two typical socialization settings for preschoolers (i.e., at home and at school). Besides, Previous study showed that positive school climate (Finch et al., 2023), living in multi-stressed families (e.g., single-mother families) (Wang et al., 2020) were associated with decrease in both externalizing and internalizing problems. This study has not yet considered these variables, so caution is needed when examining these relationships. Future research will be needed to clarify further the impact of family structure and environmental factors. Finally, current study only examined the moderating role of surgency, one of components of temperament. Other components of temperament, such as negative emotions, may also have a moderating role between CU behaviors and conduct problems. Given that negative emotions consist of components that are tend to be inhibited (e.g., fear) and other components that are tend to be provoked to action (e.g., anger or frustration) (Rothbart, 2012), different subcomponents of negative emotions may play a moderating role in different directions between CU behaviors and conduct problems. Future research can further examine the mechanisms of negative emotion subcomponents between CU behaviors and conduct problems.

Despite these limitations, the current study has multiple strengths. First, previous studies examined the relationships between CU behaviors and conduct problems in clinically referred samples. The present results from Chinese typically developing sample enriched the research on CU behaviors of preschoolers. Secondly, this study was conducted with 2,154 preschool children, and a larger sample size can increase the ecological validity of the findings. Moreover, the current study was the first to examine whether surgency play a moderating role in the relationship between CU behaviors and conduct problems in preschoolers, which provided empirical support for an in-depth exploration of potential pathways of CU behaviors and conduct problems in preschoolers, leading to the development of individualized intervention programs.

## Conclusion

This study revealed that CU behavior significantly predicted conduct problems among Chinese preschoolers. Besides, surgency played a moderating role between CU behaviors and conduct problems. Specially, CU behaviors were positively associated with conduct problems with a stronger effect found for high levels of surgency. Additionally, we found gender moderated the relationship of CU behaviors and conduct problems. Specially, CU behaviors had a positive association with conduct problems, with a stronger effect found for boys.

## Data availability statement

The data and materials used during the current study are available from the corresponding author (YL) on reasonable request.

## Ethics statement

The studies involving humans were approved by Shanghai Institute of Early Childhood Education, Shanghai Normal University. The studies were conducted in accordance with the local legislation and institutional requirements. Written informed consent for participation in this study was provided by the participants’ legal guardians/next of kin.

## Author contributions

JZ: Conceptualization, Data curation, Formal analysis, Funding acquisition, Investigation, Methodology, Project administration, Resources, Software, Supervision, Validation, Visualization, Writing – original draft, Writing – review & editing. XS: Conceptualization, Data curation, Formal analysis, Funding acquisition, Investigation, Methodology, Project administration, Resources, Software, Supervision, Validation, Visualization, Writing – original draft, Writing – review & editing. ZL: Writing – review & editing, Conceptualization, Formal analysis, Data curation, Investigation, Methodology, Project administration. YL: Conceptualization, Data curation, Formal analysis, Funding acquisition, Investigation, Methodology, Project administration, Resources, Software, Supervision, Validation, Visualization, Writing – original draft, Writing – review & editing.

## References

[ref1] Aiken L. S. West S. G. (1991). Multiple regression: Testing and interpreting interactions. Thousand Oaks, CA: Sage Publications, Inc.

[ref2] Bai R. Yan R. Wang Q. Li Y. Xing S. (2022). The relationships between executive functions and problem behavior: situational specificity and gender differences. Psychol. Dev. Educ. 38, 35–44. doi: 10.16187/j.cnki.issn1001-4918.2022.01.05

[ref3] Behrendt H. F. Wade M. Bayet L. Nelson C. A. Bosquet Enlow M. (2020). Pathways to social-emotional functioning in the preschool period: the role of child temperament and maternal anxiety in boys and girls. Dev. Psychol. 32, 961–974. doi: 10.1017/S0954579419000853, PMID: 31345275 PMC6982599

[ref4] Berdan L. E. Keane S. P. Calkins S. D. (2008). Temperament and externalizing behavior: social preference and perceived acceptance as protective factors. Dev. Psychol. 44, 957–968. doi: 10.1037/0012-1649.44.4.957, PMID: 18605827 PMC2773664

[ref5] Berkout O. V. Young J. N. Gross A. M. (2011). Mean girls and bad boys: recent research on gender differences in conduct disorder. Aggress. Violent Behav. 16, 503–511. doi: 10.1016/j.avb.2011.06.001

[ref6] Blair R. J. R. (1995). A cognitive developmental approach to morality: investigating the psychopath. Cognition 57, 1–29. doi: 10.1016/0010-0277(95)00676-P, PMID: 7587017

[ref7] Caiozzo C. Houston J. P. Grych J. H. (2016). Predicting aggression in late adolescent romantic relationships: a short-term longitudinal study. J. Adolesc. 53, 237–248. doi: 10.1016/j.adolescence.2016.10.012 27816698

[ref8] Calkins S. D. Propper C. B. Mills-Koonce W. R. (2013). A biopsychosocial perspective on parenting and developmental psychopathology. Dev. Psychopathol. 25, 1399–1414. doi: 10.1017/s0954579413000680, PMID: 24342847

[ref9] Cao X. Somerville M. P. Allen J. L. (2023). Teachers' perceptions of the school functioning of Chinese preschool children with callous-unemotional traits and disruptive behaviors. Teach. Teach. Educ. 123:103990. doi: 10.1016/j.tate.2022.103990

[ref10] Cicchetti D. (2014). Illustrative developmental psychopathology perspectives on precursors and pathways to personality disorder: commentary on the special issue. J. Per. Disord. 28, 172–179. doi: 10.1521/pedi.2014.28.1.172, PMID: 24344897

[ref11] Davies W. J. (2014). Sex differences in attention deficit hyperactivity disorder: candidate genetic and endocrine mechanisms. Front. Neuroendocrinol. 35, 331–346. doi: 10.1016/j.yfrne.2014.03.003, PMID: 24680800

[ref12] del Puerto-Golzarri N. Azurmendi A. Carreras M. R. Muñoz J. M. Braza P. Vegas O.. (2022). The moderating role of surgency, behavioral inhibition, negative emotionality and effortful control in the relationship between parenting style and children’s reactive and proactive. Aggression. Child 9:104. doi: 10.3390/children9010104, PMID: 35053729 PMC8774234

[ref13] Deng Q. Ding C. Liu M. Deng J. Wang M. (2016). Psychometric properties of the inventory of callous-unemotional in preschool students. J. Clin. Psychol. 24:663666. doi: 10.16128/j.cnki.1005-3611.2016.04.020

[ref14] Díaz-Vázquez B. López-Romero L. Romero E. (2024). Emotion recognition deficits in children and adolescents with psychopathic traits: a systematic review. Clin. Child. Fam. Psychol. Rev. 27, 165–219. doi: 10.1007/s10567-023-00466-z, PMID: 38240937 PMC10920463

[ref15] Domínguez-Álvarez B. Romero E. López-Romero L. Isdahl-Troye A. Wagner N. L. Waller R. (2021). A cross-sectional and longitudinal test of the low sensitivity to threat and affiliative reward (STAR) model of callous-unemotional traits among Spanish preschoolers. Res. Child. Adolesc. Psychopathol. 49, 877–889. doi: 10.1007/s10802-021-00785-1, PMID: 33624154

[ref16] Du Y. Kou J. Coghill D. (2008). The validity, reliability and normative scores of the parent, teacher and self-report versions of the strengths and difficulties questionnaire in China. Child Adolesc. Psychiatry Ment. Health 2, 1–15. doi: 10.1186/1753-2000-2-8, PMID: 18445259 PMC2409296

[ref17] Eagly A. H. (1987). Sex differences in social behavior: A social-role interpretation. Hillsdale, NJ: Erlbaum.

[ref18] Fang J. Wang X. Yuan K. Wen Z. Yu X. Zhang G. (2020). Callous-unemotional traits and cyberbullying perpetration: the mediating role of moral disengagement and the moderating role of empathy. Pers. Individ. Dif. 157:109829. doi: 10.1016/j.paid.2020.109829

[ref19] Finch J. E. Akhavein K. Patwardhan I. Clark C. A. (2023). Teachers' self-efficacy and perceptions of school climate are uniquely associated with students' externalizing and internalizing behavior problems. J. Appl. Dev. Psychol. 85:101512. doi: 10.1016/j.appdev.2023.101512, PMID: 36817721 PMC9937514

[ref20] Frick P. J. Cornell A. H. Bodin S. D. Dane H. E. Barry C. T. Loney B. R. (2003). Callous-unemotional traits and developmental pathways to severe conduct problems. Dev. Psychol. 39, 246–260. doi: 10.1037/0012-1649.39.2.246, PMID: 12661884

[ref21] Frick P. J. Liddle J. A. Thornton L. M. Kahn R. E. (2014). Can callous-unemotional traits enhance the understanding, diagnosis, and treatment of serious conduct problems in children and adolescents? A comprehensive review. Psychol Bull. 140, 1–57. doi: 10.1037/a0033076, PMID: 23796269

[ref22] Frick P. J. White S. F. (2008). Research review: the importance of callous-unemotional traits for developmental models of aggressive and antisocial behavior. J. Child Psychol. Psychiatry 49, 359–375. doi: 10.1111/j.1469-7610.2007.01862.x, PMID: 18221345

[ref23] Fung A. L. C. Gao Y. Raine A. (2009). The utility of the child and adolescent psychopathy construct in Hong Kong, China. J. Clin. Child Adolesc. Psychol. 39, 134–140. doi: 10.1080/15374410903401138 20390805

[ref24] Garon-Carrier G. Pascuzzo K. Gaudreau W. Lemelin J. P. Déry M. (2022). Maternal functioning and child's externalizing problems: temperament and sex-based driven effects. Front. Psychol. 13:874733. doi: 10.3389/fpsyg.2022.874733, PMID: 35664135 PMC9157281

[ref25] Goodman R. (1997). The strengths and difficulties questionnaire: a research note. J. Child Psychol. Psychiatry 38, 581–586. doi: 10.1111/j.1469-7610.1997.tb01545.x 9255702

[ref27] Hancock G. R. Mueller R. O. (2010). The reviewer’s guide to quantitative methods in the social sciences. New York, NY: Routledge.

[ref28] Hayes A. F. (2018). Introduction to mediation, moderation, and conditional process analyses: A regression based approach (2nd Edn). New York: Guiford Press.

[ref29] Hill N. E. Castellino D. R. Lansford J. E. Nowlin P. Dodge K. A. Bates J. E.. (2004). Parent academic involvement as related to school behavior, achievement, and aspirations: demographic variations across adolescence. Child Dev. 75, 1491–1509. doi: 10.1111/j.1467-8624.2004.00753.x, PMID: 15369527 PMC2765127

[ref31] Kim H. Chang H. (2019). Longitudinal association between children’s callous-unemotional traits and social competence: child executive function and maternal warmth as moderators. Front. Psychol. 10, 379–391. doi: 10.3389/fpsyg.2019.00379 30873083 PMC6403163

[ref32] Kimonis E. R. Fleming G. Briggs N. Brouwer-French L. Frick P. J. Hawes D. J.. (2019). Parent-child interaction therapy adapted for preschoolers with callous-unemotional traits: an open trial pilot study. J. Clin. Child Adolesc. Psychol. 48, S347–S361. doi: 10.1080/15374416.2018.1479966, PMID: 29979887

[ref9001] Leff S. S. Paskewich B. S. Fu R. Waasdorp T. E. (2024). “School-based aggression and bullying prevention program implementation in real-world conditions,” in Cyber and face-to-face aggression and bullying among children and adolescents. ed. Fung A. L. C. (London: Routledge), 120–131.

[ref34] Liu X. Liu S. Mo B. Li D. Fu R. (2020). Extroversion and aggressive behavior in early childhood: moderating effects of self-control and maternal warmth. Psychol. Dev. Educ. 36, 538–544. doi: 10.16187/j.cnki.issn1001-4918.2020.05.04

[ref35] Longman T. Hawes D. J. Kohlhoff J. (2016). Callous-unemotional traits as markers for conduct problem severity in early childhood: a meta-analysis. Child. Psychiat. Hum. D. 47, 326–334. doi: 10.1007/s10578-015-0564-9, PMID: 26123709

[ref36] Ma Y. Xing X. Zhang M. (2022). Parental rejection and school-aged children’s externalizing behavior problems in China: the roles of executive function and callous-unemotional traits. Child. Psychiat. Hum. D. 55, 152–163. doi: 10.1007/s10578-022-01397-6 35789449

[ref37] Miller J. B. (1976). Toward a new psychology of women. Boston, MA: Beacon Press.

[ref38] Miller J. B. Stiver I. P. (1997). The healing connection: How women form relationship in therapy and in life. Boston, MA: Beacon Press.

[ref39] Obando D. Hill J. Sharp H. Pickles A. Fisher L. Wright N. (2022). Synergy between callous-unemotional traits and aggression in preschool children: cross-informant and cross-cultural replication in the UK Wirral child health and development study, and the Colombian La Sabana parent-child study. Dev. Psychopathol. 34, 1079–1087. doi: 10.1017/S0954579420002114, PMID: 33752771

[ref40] Paz Y. Orlitsky T. Roth-Hanania R. Zahn-Waxler C. Davidov M. (2021). Predicting externalizing behavior in toddlerhood from early individual differences in empathy. J. Child Psychol. Psychiatry 62, 66–74. doi: 10.1111/jcpp.13247, PMID: 32645218

[ref41] Putnam S. P. Rothbart M. K. (2006). Development of short and very short forms of the children’s behavior questionnaire. J. Pers. Assess. 87, 102–112. doi: 10.1207/s15327752jpa8701_09 16856791

[ref42] Rehder P. D. Mills-Koonce W. R. Willoughby M. T. Garrett-Peters P. Wagner N. J. (2017). Emotion recognition deficits among children with conduct problems and callous-unemotional behaviors. Early Child Res. Q. 41, 174–183. doi: 10.1016/j.ecresq.2017.07.007, PMID: 34113059 PMC8188849

[ref43] Rothbart M. K. (2012). Becoming who we are: Temperament and personality in development. New York, NY: Guilford Press.

[ref44] Rothbart M. K. Ahadi S. A. Hershey K. L. Fisher P. (2001). Investigations of temperament at three to seven years: the children’s behavior questionnaire. Child Dev. 72, 1394–1408. doi: 10.1111/1467-8624.00355, PMID: 11699677

[ref46] Scheper F. Y. Majdandžić M. van de Ven P. M. Jansen L. Doreleijers T. A. Schuengel C.. (2017). Temperament traits and psychopathology in young clinically referred children compared to a general population sample. Child. Psychiat. Hum. D. 48, 841–850. doi: 10.1007/s10578-016-0708-6, PMID: 28097446 PMC5680369

[ref9003] Sharp C. Van Goozen S. Goodyer I. (2006). Children’s subjective emotional reactivity to affective pictures: gender differences and their antisocial correlates in an unselected sample of 7–11‐year‐olds. J. Child Psychol. Psychiatry 47, 143–150. doi: 10.1111/j.1469-7610.2005.01464.x 16423145

[ref9004] Simon P. Nader-Grosbois N. (2021). Preschoolers’ empathy profiles and their social adjustment. Front. psychol. 12:782500. doi: 10.3389/fpsyg.2021.782500 34956001 PMC8704132

[ref47] Sng K. I. Hawes D. J. Hwang S. Allen J. L. Fung D. S. (2020). Callous-unemotional traits among children and adolescents in Asian cultures: a systematic review. J. Cross-Cult. Psychol. 51, 576–596. doi: 10.1177/0022022120944475

[ref48] Song J. H. Waller R. Hyde L. W. Olson S. L. (2016). Early callous-unemotional behavior, theory-of-mind, and a fearful/inhibited temperament predict externalizing problems in middle and late childhood. J. Abnorm. Child Psychol. 44, 1205–1215. doi: 10.1007/s10802-015-0099-3, PMID: 26582182 PMC4937620

[ref49] Wagner N. J. Bowker J. C. Rubin K. H. (2020). Associations between callous-unemotional traits and peer-rated social-behavioral outcomes in elementary and middle school. J. Abnorm. Child Psychol. 48, 757–769. doi: 10.1007/s10802-020-00636-5, PMID: 32185609

[ref50] Wagner N. J. Hastings P. D. Rubin K. H. (2018). Callous-unemotional traits and autonomic functioning in toddlerhood interact to predict externalizing behaviors in preschool. J. Abnorm. Child Psychol. 46, 1439–1450. doi: 10.1007/s10802-017-0374-6, PMID: 29188411

[ref51] Waller R. Dishion T. J. Shaw D. S. Gardner F. Wilson M. N. Hyde L. W. (2016). Does early childhood callous-unemotional behavior uniquely predict behavior problems or callous-unemotional behavior in late childhood? Dev. Psychol. 52, 1805–1819. doi: 10.1037/dev0000165, PMID: 27598253 PMC5083155

[ref9002] Waller R. Gardner F. Hyde L. W. Shaw D. S. Dishion T. J. Wilson M. N. (2012). Do harsh and positive parenting predict parent reports of deceitful‐callous behavior in early childhood? J. Child Psychol. Psychiatry 53, 946–953. doi: 10.1111/j.1469-7610.2012.02550.x 22490064 PMC3454481

[ref52] Waller R. Hyde L. W. Baskin-Sommers A. R. Olson S. L. (2017). Interactions between callous unemotional behaviors and executive function in early childhood predict later aggression and lower peer-liking in late-childhood. J. Abnorm. Child Psychol. 45, 597–609. doi: 10.1007/s10802-016-0184-2, PMID: 27418255 PMC5944342

[ref53] Waller R. Hyde L. W. Grabell A. S. Alves M. L. Olson S. L. (2015). Differential associations of early callous-unemotional, oppositional, and ADHD behaviors: multiple domains within early-starting conduct problems? J. Child Psychol. Psychiatry 56, 657–666. doi: 10.1111/jcpp.12326, PMID: 25251938 PMC4937618

[ref54] Waller R. Wagner N. (2019). The sensitivity to threat and affiliative reward (STAR) model and the development of callous-unemotional traits. Neurosci. Biobehav. Rev. 107, 656–671. doi: 10.1016/j.neubiorev.2019.10.005, PMID: 31618611

[ref55] Waller R. Wagner N. J. Barstead M. G. Subar A. Petersen J. L. Hyde J. S.. (2020). A meta-analysis of the associations between callous-unemotional traits and empathy, prosociality, and guilt. Clin. Psychol. Rev. 75:101809. doi: 10.1016/j.cpr.2019.101809, PMID: 31862383

[ref56] Wang D. Choi J. K. Shin J. (2020). Long-term neighborhood effects on adolescent outcomes: mediated through adverse childhood experiences and parenting stress. J. Youth Adolesc. 49, 2160–2173. doi: 10.1007/s10964-020-01305-y 32804295

[ref57] Wang G. Liu Z. B. (2010). What collective? Collectivism and relationalism from a Chinese perspective. Chin. J. Commun. 3, 42–63. doi: 10.1080/17544750903528799

[ref58] Wang Y. Zhang W. Li D. P. Li D. L. Zhang X. (2012). Temperament and adolescent tobacco and alcohol use: a test of interaction effects. Psychol. Dev. Educ. 28, 292–300. doi: 10.16187/j.cnki.issn1001-4918.2012.03.005

[ref59] Willoughby M. T. Mills-Koonce W. R. Gottfredson N. C. Wagner N. J. (2014). Measuring callous unemotional behaviors in early childhood: factor structure and the prediction of stable aggression in middle childhood. J. Psychopathol. Behav. Assess. 36, 30–42. doi: 10.1007/s10862-013-9379-9, PMID: 24729655 PMC3979638

[ref61] Zhang J. Li W. Zhang H. Wilson A. Shuai L. Xia W.. (2021). Callous-unemotional traits in Chinese preschool children with attention-deficit/hyperactivity disorder. Child Adolesc. Psychiatry Ment. Health 15, 35–39. doi: 10.1186/s13034-021-00388-0, PMID: 34246300 PMC8272896

[ref60] Zhang L. Chen Y. Hong X. Zhao M. Fan H. Liu S. (2023). The relationship between callous-unemotional traits and school bullying of junior high school students: a moderated mediation model. Psychol. Dev. Educ. 39, 266–275. doi: 10.16187/j.cnki.issn1001-4918.2023.02.13

[ref62] Zhou Q. Main A. Wang Y. (2010). The relations of temperamental effortful control and anger/frustration to Chinese children’s academic achievement and social adjustment: a longitudinal study. J. Educ. Psychol. 102, 180–196. doi: 10.1037/a0015908

[ref63] Zhu J. Shu X. Dong X. Li Y. (2023b). Types and characteristics of callous-unemotional traits in preschool children and their school adjustment based on latent profile analysis. J. Clin. Psychol. 31, 1214–1219+1224. doi: 10.16128/j.cnki.1005-3611.2023.05.037

[ref64] Zhu J. Shu X. Zou S. Li Y. (2023a). Reliability and validity of the inventory of callous-unemotional traits (teacher version) to Chinese preschoolers. J. Clin. Psychol. 31, 620–624. doi: 10.16128/j.cnki.1005-3611.2023.03.022

